# Identification of CYP 2A6 inhibitors in an effort to mitigate the harmful effects of the phytochemical nicotine

**DOI:** 10.20517/2394-4722.2020.143

**Published:** 2021-04-14

**Authors:** Navneet Goyal, Jayalakshmi Sridhar, Camilla Do, Melyssa Bratton, Shahensha Shaik, Quan Jiang, Maryam Foroozesh

**Affiliations:** 1 Department of Chemistry, Xavier University of Louisiana, New Orleans, LA 70125, USA.; 2 Cell and Molecular Biology and Bioinformatic Core, College of Pharmacy, Xavier University of Louisiana, New Orleans, LA 70125, USA.

**Keywords:** Phytochemicals, cytochrome P450 enzymes, tobacco smoking, nicotine

## Abstract

**Aim:**

In this study, our goal was to study the inhibition of nicotine metabolism by P450 2A6, as a means for reduction in tobacco use and consequently the prevention of smoking-related cancers. Nicotine, a phytochemical, is an addictive stimulant, responsible for the tobacco-dependence in smokers. Many of the other phytochemicals in tobacco, including polycyclic aromatic hydrocarbons, N-nitrosamines, and aromatic amines, are potent systemic carcinogens. Tobacco smoking causes about one of every five deaths in the United States annually. Nicotine plasma concentration is maintained by the smokers’ smoking behavior within a small range. Nicotine is metabolized by cytochrome P450s 2A6 and 2A13 to cotinine. This metabolism causes a decrease in nicotine plasma levels, which in turn leads to increased tobacco smoking, and increased exposure to the tobacco carcinogens.

**Methods:**

Using the phytochemical nicotine as a lead structure, and taking its interactions with the P450 2A6 binding pocket into consideration, new pyridine derivatives were designed and synthesized as potential selective mechanism-based inhibitors for this enzyme.

**Results:**

The design and synthesis of two series of novel pyridine-based compounds, with varying substituents and substitution locations on the pyridine ring, as well as their inhibitory activities on cytochrome P450 2A6 and their interactions with its active site are discussed here. Substitutions at position 3 of the pyridine ring with an imidazole or propargyl ether containing group showed the most optimal interactions with the P4502A6 active site.

**Conclusion:**

The pyridine compounds with an imidazole or propargyl ether containing substituent on position 3 were found to be promising lead compounds for further development. Hydrogen-bonding interactions were determined to be crucial for effective binding of these molecules within the P450 2A6 active site.

## INTRODUCTION

Phytochemicals are plant-produced natural products, and are commonly found in human and animal diets as well as in plant-based medications and natural remedies^[1–3]^. While most of these chemicals have basic nutritional value, some have preventive, therapeutic or toxic effects. Phytochemicals can play a preventive role in carcinogenesis in multiple ways; as primary preventive agents that can be used to prevent the advent of cancer; as secondary agents that can prevent progression of cancer as in the case of premalignant lesions; and as tertiary agents that can prevent the recurrence of cancer^[4,5]^. Some examples of such phytochemicals are capsaicin from chili pepper^[6,7]^, polyphenols from green tea, fruits, and vegetables^[8,9]^, carotenoids such as lycopene found in fruits^[10,11]^, cucurbitacin B from Chinese medicinal plants^[12–14]^, isoflavones from legumes^[15,16]^, etc. Phytochemicals have also been shown to modulate multiple mechanisms in cancer, resulting in their anti-cancer activities^[5,17–21]^. Phytochemicals are known to have similar preventive and/or therapeutic effects on many other diseases^[10,11,16,19,22]^.

Phytochemicals are often toxins produced by plants as a defense mechanism against disease-causing organisms or herbivorous animals. Certain herbs and plants routinely used by humans, contain phytotoxins with carcinogenic, teratogenic and/or endocrine influencing activities^[1]^. While some of these phytochemicals are direct-acting toxins, others, such as procarcinogenic agents, need metabolic activation. Certain procarcinogenic alkenylbenzenes, pyrrolizidine alkaloids, ptaquiloside, aristolochic acids, and furanocoumarins are known DNA-alkylating agents^[3,22–25]^. Metabolic activation of phytochemical procarcinogens into their ultimate carcinogenic forms by phase I and phase II enzymes has been well established^[26,27]^. Cytochrome P450 enzymes, a superfamily of Phase I enzymes, metabolize endogenous and xenobiotic compounds including phytochemicals, through monooxygenation reactions^[26–29]^.

Nicotine, a phytotoxin present in many plants and vegetables, including tobacco, and in much smaller concentrations in potatoes, tomatoes, eggplants, and green peppers, is primarily metabolized by human liver P450 2A6, and to a smaller extent by human lung P450 2A13. The metabolism of nicotine to cotinine takes place in two steps - initial oxidation to the intermediate nicotine-△^1’(5’)^-iminium ion by either of the two P450 enzymes^[29–31]^, and further oxidation to cotinine by cytosolic aldehyde oxidase^[29,32]^. Nicotine plays a critical role in tobacco-dependence, and is the main cause of lung cancer deaths in men and women. It is estimated that more than 16 million Americans are affected by cigarette smoking, and more than 480,000 deaths per year are attributed to tobacco use^[33]^. Several strategies have been developed and implemented for cessation of cigarette smoking, such as nicotine replacement therapies alone or in combination with varenicline, a nicotinic acetylcholine receptor partial agonist^[34]^. However, only about 6% of smokers are able to overcome the addiction to nicotine^[35]^. The addiction to nicotine in smokers modulating their smoking behaviors has a direct correlation to the levels of nicotine in the blood and brain^[36–38]^. Drop of the levels of nicotine in the blood and brain due to the P450 2A6 metabolism, causes the smokers to adjust their tobacco use to maintain these levels. The impact of P450 2A6 activity on tobacco addiction is evidenced in individuals who are slow metabolizers due to P450 2A6 polymorphism, and thus smoke less^[37,39,40]^.

Inhibition of P450 2A6, which is known to be well tolerated by humans^[41]^, is emerging as the most promising strategy for smoking cessation and treatment of tobacco-dependence. Development of P450 2A6-specific inhibitors is currently pursued by many research groups^[42–47]^. Recent developments in this field include the use of bioelectrochemical platforms that use the “molecular lego” approach^[48,49]^. In the study reported by Castrignanò *et al*^[50]^, genetically-fused P450 2A6 with *Desulfovibrio* vulgaris flavodoxin (FLD) module is used for investigating the inhibitory effects of coumarins and nicotine. Such an approach could improve and hasten the process of P450 enzyme inhibitor development. The development of mimicking agents with structural similarities to nicotine through the modification of its pyridine ring substituents, by our research group and the Cashman Research Group^[51–54]^, has led to potent (with low micromolar IC_50_ values) inhibitors of P450 2A6^[42,51,54]^. For this study, we have identified two series of such pyridine-based P450 2A6 inhibitors.

## METHODS

**Synthesis of 3-((prop-2-yn-1-yloxy)methyl)pyridine (6):** 3-Hydroxymethylpyridine (1.0 eq) in tetrahydrofuran (THF) was added dropwise to a cooled (0 °C) suspension of sodium hydride (NaH, 95%, 2.1 eq) in dry THF. After 20 min, propargyl bromide (80% solution in toluene, 2.0 eq) was added slowly. The reaction mixture was heated at 50 °C overnight. It was then allowed to cool to room temperature before careful quenching by the addition of water. The crude was extracted with ethyl acetate. The organic layer was washed with brine and dried over anhydrous sodium sulfate (Na_2_SO_4_). The solvent was removed under vacuum. The residue was then purified by silica gel column chromatography using hexane: ethyl acetate (1:3) as eluent to afford the desired product.

**Compound 6:** (96% yield; brown liquid) GC-MS showed > 99% purity. m/z: 146, 108, 92, 80, 65, 51. ^1^HNMR (CDCl_3_, 300 MHz) δ = 2.45 (t, *J* = 2.4 Hz, 1H), 3.42 (s, 2H), 4.51 (s, 2H), 7.32 (m, 1H), 7.62 (m, 1H), and 8.55 (m, 1H). ^13^C NMR (CDCl_3_, 75 MHz) δ = 57.7, 69.1, 75.3, 79.3, 123.5, 133.0, 135.8, 149.5, and 149.6.

**Syntheses of 4-(prop-2-yn-1-yloxy)pyridine (2), 2-(prop-2-yn-1-yloxy)pyridine (3) and 4-(3-(prop-2-yn-1-yloxy)propyl)pyridine (5)** were achieved using the same procedure.

**Compound 2:** (82% yield; white solid) GC-MS showed > 98% purity, m/z: 133.1, 104.1, 78.0, 52.0. ^1^HNMR (CDCl_3_, 300 MHz) δ = 2.63 (t, *J* = 2.56 Hz, 1H), 4.50 (d, *J* = 2.6 Hz, 2H), 6.35 (d, *J* = 7.3 Hz, 2H), and 7.42 (d, *J* = 7.6 Hz, 2H). ^13^C NMR (CDCl_3_, 75 MHz) δ = 45.5, 75.7, 77.3, 118.8, 139.6, and 179.1.

**Compound 3:** (76% yield; white solid) GC-MS showed > 99% purity, m/z: 133.1, 104.1, 78.0, 52.0. ^1^HNMR (CDCl_3_, 300 MHz) δ = 2.48 (t, *J* = 2.4 Hz, 1H), 4.72 (d, *J* = 2.3 Hz, 2H), 6.22 (t, *J* = 6.1 Hz, 1H), 6.54 (d, *J* = 5.2 Hz, 1H), 7.36 (d, *J* = 2.8 Hz, 1H), and 7.63 (d, *J* = 4.6 Hz, 1H). ^13^C NMR (CDCl_3_, 75 MHz) δ = 37.5, 75.3, 77.3, 106.2, 120.1, 136.5, 139.9, and 161.6.

**Compound 5:** (67% yield; yellow liquid) GC-MS showed > 98% purity. m/z: 174, 158, 145, 118, 110, 93. ^1^HNMR (CDCl_3_, 300 MHz) δ = 2.0 (m, 2H), 2.42(t, *J* = 2.6 Hz, 1H), 2.89 (t, *J* = 7.4 Hz, 2H), 3.56 (t, *J* = 6.3 Hz, 2H), 4.14 (d, *J* = 2.3 Hz, 2H), 7.15 (m, 2H), 7.60 (m, 1H), and 8.52 (m, 1H). ^13^C NMR (CDCl_3_, 75 MHz) δ = 30.1, 31.6, 58.2, 68.8, 74.6, 77.1, 124.1, 149.8, and 150.8.

**Synthesis of 3-((1H-imidazol-1-yl)methyl)pyridine (4):** The solution of 3-chloromethylpyridine hydrochloride salt (1.0 eq) and imidazole (10.0 eq) in dimethylformamide (DMF) (10 mL) was heated at 100 °C until all the salt starting material was consumed (~3 h). The light brown solution was cooled down to room temperature, and the DMF was removed under vacuum. The residue was dissolved in dichloromethane (DCM), and washed with water. The DCM layer was dried over anhydrous Na_2_SO_4_. The solvent was evaporated under vacuum to afford the desired product.

**Compound 4:** (14% yield; brown solid) GC-MS showed > 99% purity. m/z: 159, 132, 92. 65. ^1^HNMR (CDCl_3_, 300 MHz) δ = 5.10 (s, 2H), 6.88 (s, 1H), 7.00 (s, 1H), 7.31 (m, 2H), 7.65 (s, 1H), 8.46 (m, 1H), and 8.56 (m, 1H). 13C NMR (CDCl_3_, 75 MHz) δ = 48.3, 119.3, 123.9, 130.0, 135.0, 137.3, 148.7, and 149.7.

**Syntheses of 3-(3-(2-methyl-1H-imidazol-1-yl))propyl]pyridine (1) and 3-[(2-methyl-1H-imidazol-1-yl)methyl] pyridine (7)** were achieved using the same procedure.

**Compound 1:** (86% yield; yellow oil) GC-MS showed > 99% purity. m/z: 201, 186, 118, 96, 55. ^1^HNMR (CDCl_3_, 300 MHz) δ = 2.05 (m, 2H), 2.32 (s, 3H), 2.62 (t, *J* = 7.7 Hz, 2H), 3.85 (t, *J* = 7.4 Hz, 2H), 6.80 (d, *J* = 1.0 Hz, 1H), 6.90 (d, *J* = 1.1 Hz, 1H), 7.22 (m, 1H), 7.45 (m, 1H), and 8.45 (m, 2H). ^13^C NMR (CDCl_3_, 75 MHz) δ = 12.6, 29.3, 31.3, 44.7, 118.7, 120.9, 123.1, 126.6, 135.3, 135.6, 143.8, 147.3, and 149.3.

**Compound 7:** (15% yield; brown oil) GC-MS showed > 98% purity. m/z: 173, 146, 92, 65. ^1^HNMR (CDCl_3_, 300 MHz) δ = 2.35 (s, 3H), 5.0 (s, 2H), 6.84 (s, 1H), 6.97 (s, 1H), 7.31 (m, 2H), 8.46 (m, 1H), and 8.56 (m, 1H). ^13^C NMR (CDCl_3_, 75 MHz) δ = 13.1, 47.4, 119.8, 123.9, 127.8, 132.1, 132.4, 144.8, 148.4, and 149.6.

### P450 2A6 inhibition assay

Cytochrome P450 2A6 (Cyp2A6) activity was determined using the Vivid CYP450 Screening Kit (Life Technologies, catalog #PV6140) according to the manufacturer’s instructions. Briefly, a master pre-mix containing baculosomes and regeneration system was prepared using 0.5× Vivid reaction buffer II. The test compounds were dissolved in dimethyl sulfoxide (DMSO) at a concentration of 100 mM. From the stock solutions, each compound was serially diluted in 0.5× Vivid reaction buffer II to make working stocks of 100 μM, 50 μM, 25 μM and so on up to 10 dilutions. It is essential to dilute the DMSO at least 1000-fold when making the working dilutions to prevent its interference with the enzyme activity. An initial high-throughput screening was performed at 10 μM concentration for each compound. For the dose-response curve determination, in a 96-well plate, 40 μL of the diluted solutions of each test compound were added to each well, followed by 50 μl of the master pre-mix, before incubation for 10 min at room temperature. A 10× mixture of Vivid substrate (reconstituted with acetonitrile) and NADP^+^ was then prepared. At the end of the incubation period, 10 μL of this solution was added to each well to start the reaction. After 2 hours of incubation in the dark at room temperature, the plate was read at 415 nm on a plate reader (Synergy H1, Biotek). For a positive inhibition control, tranylcypromine (Sigma-Aldrich, cat. #P8511) was used at a final concentration of 100 μM. For a negative (no inhibitor) control, a 1:1000 dilution of pure DMSO in 0.5× Vivid reaction buffer was used. Each concentration in the dose-response curve was set up in triplicates, and each data point was the average of triplicate wells. The % inhibition was calculated using the following equation.

$$\%\text{Inhibition}=\left(1-\frac{X-B}{A-B}\right)*100$$

where X is the fluorescence observed in presence of the test compound; A is the fluorescence observed in the absence of an inhibitor (no inhibitor control); and B is the fluorescence observed for the positive control. For graphing purposes, percent inhibition *vs*. anti-log[drug concentration] was plotted. A logistic sigmoidal model was used to fit the data and obtain IC_50_ values using Graphpad Prism software.

### Docking studies

Docking studies were performed using methods previously published^[55–57]^. The coordinates of the reported X-ray crystal structure of P450 2A6 bound to nicotine^[58]^ (4EJJ.pdb) were downloaded from the Protein Data Bank website (http://www.rcsb.org/), and used for the docking studies. Heme was considered as part of the receptor for docking purposes. Standard force fields were used for the compounds. MOE energy minimization method was used using the MOE software platform from the ChemComp group.

## RESULTS

Nicotine, the main addictive ingredient of tobacco products, is mainly (70%−80%) metabolized to cotinine by P450 2A6 enzyme in the liver. Cotinine has a longer half-life but is much less active in inducing dopamine release in smokers than nicotine^[30]^. As nicotine is metabolized, maintenance of its blood plasma levels compels the smokers to modify their smoking frequency to compensate. Tobacco usage has been linked to several debilitating diseases, with lung cancer being the most common disease among smokers. Inhibition of P450 2A6 can prove to be one of the most effective strategies for smoking cessation. Our research group has been exploring several classes of compounds as possible P450 2A6 inhibitors. The use of the pyridine molecule as a scaffold for P450 2A6 inhibitors has been explored by our research group and the Cashman Research Group^[51–54]^. The incorporation of 5-membered heterocyclic rings has been pursued by both research groups. One of the distinctive features of the P450 2A6 inhibitors developed by the Cashman Group has been the use of a primary amino group as the main functional group that binds to the heme-Fe of the enzyme. In contrast, our research group is focused on (1) the incorporation of a triple bond that can lead to mechanism-based inhibition of the enzyme; and (2) the incorporation of an imidazole ring that can mimic the pyrrolidine ring of nicotine, in which the −CH of the 5-membered ring faces the heme-Fe of the enzyme.

**Figure 1.**
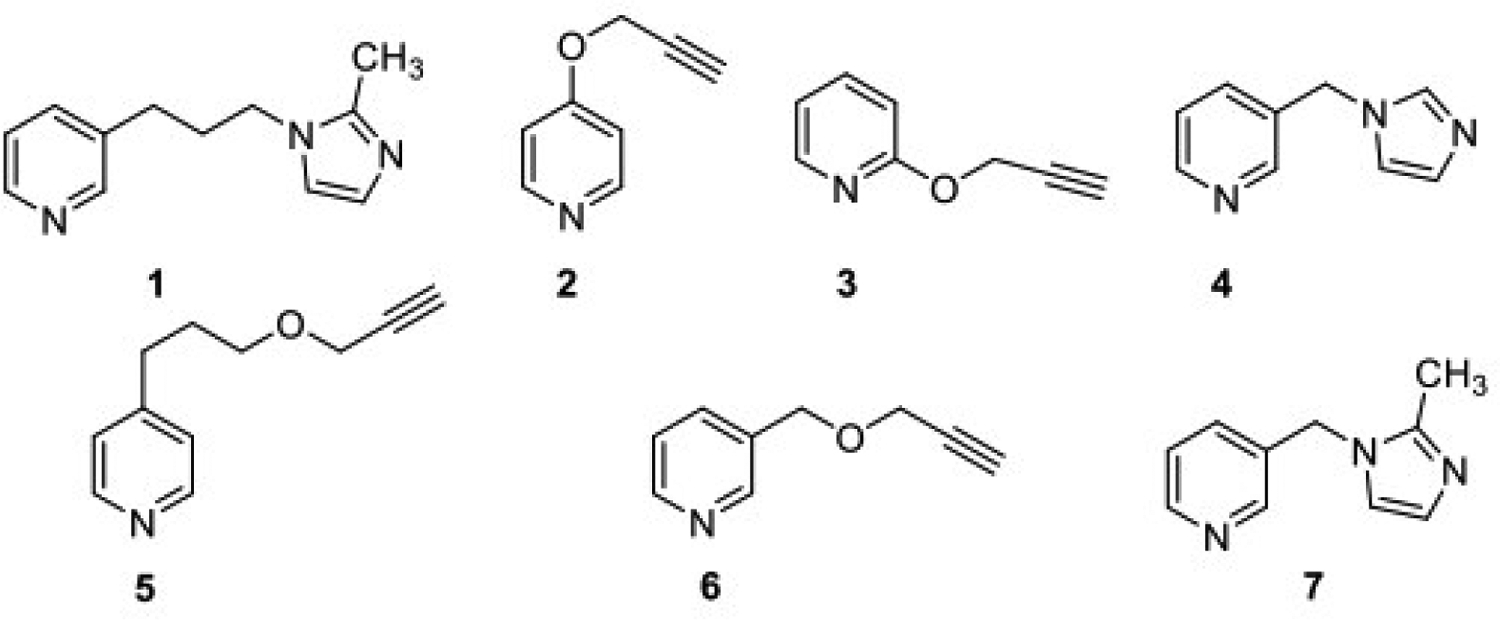
Structures of Compounds 1 to 7.

**Figure 2.**
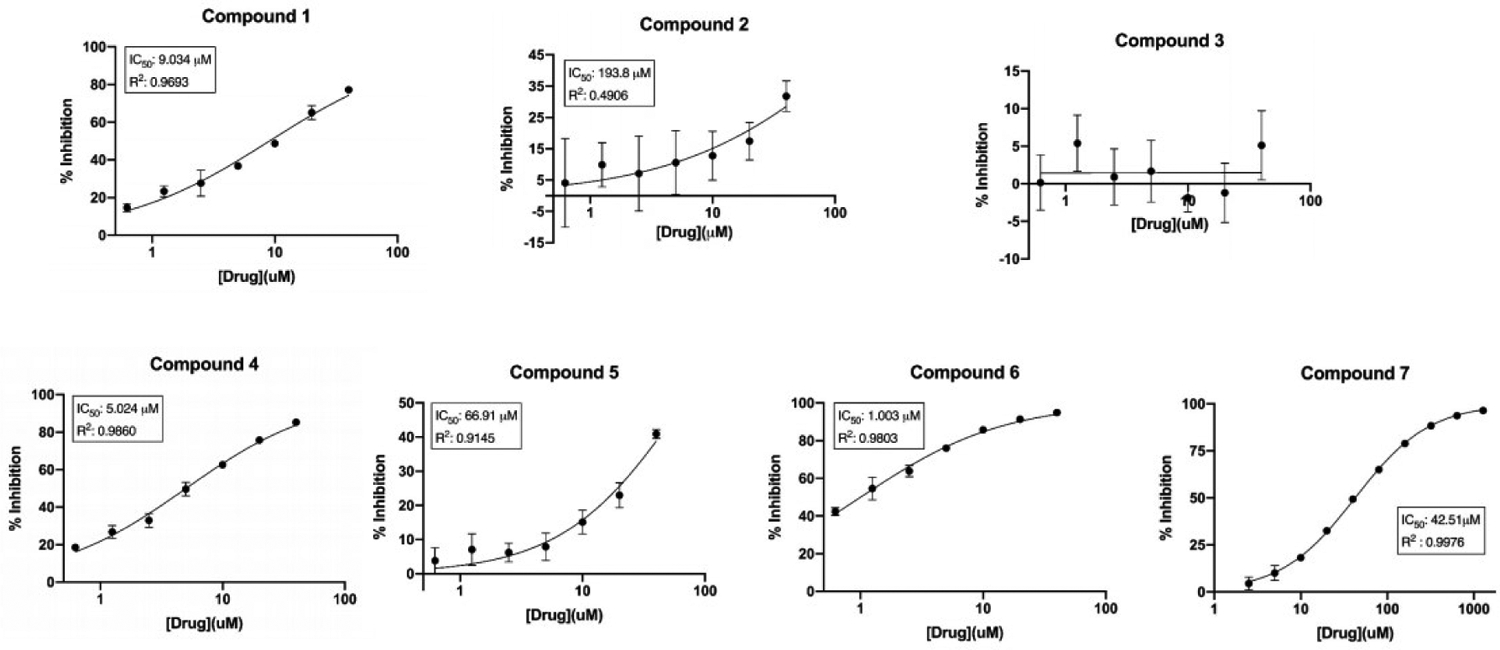
Dose-response curves for the inhibition of P450 2A6 by compounds 1 to 7. The concentrations of the compounds are represented on the X-axis as an antilog scale. The highest compound concentrations used for dose-response curves is 40 μM.

**Scheme 1.**
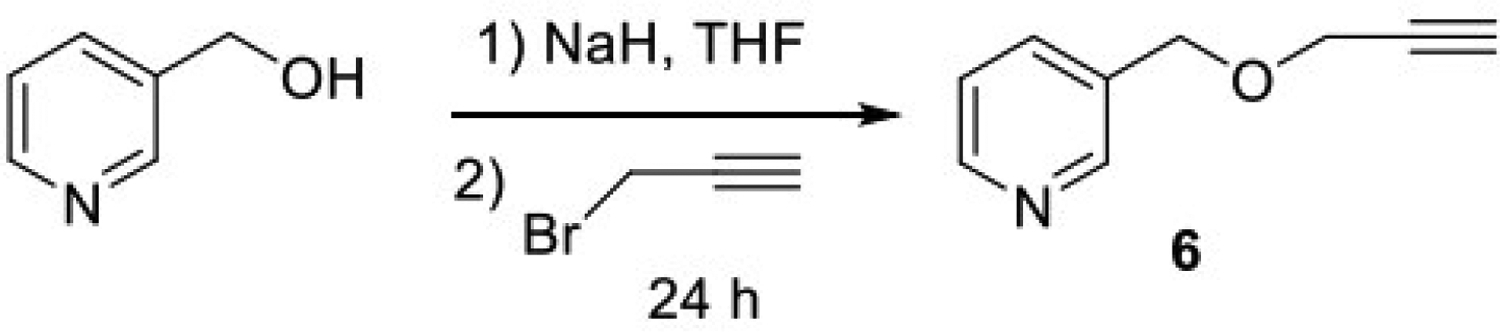
Synthesis of compounds 2, 3, 5 and 6 were accomplished using the following scheme.

**Scheme 2.**
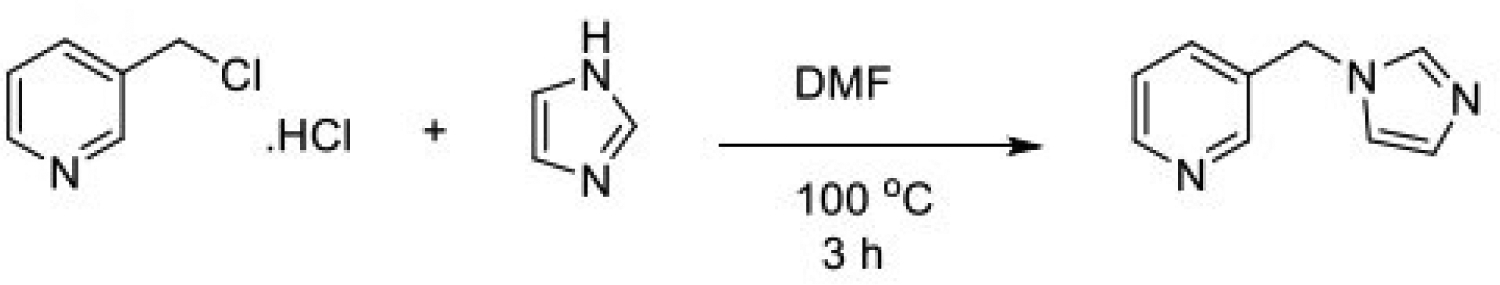
Synthesis of compounds 1, 4 and 7 were accomplished using the following scheme.

The syntheses of compounds 1 to 7 [Figure 1] were accomplished using the synthetic Schemes 1 and 2. The binding mode of nicotine to the P450 enzyme in the X-ray crystal structure (4EJJ.pdb) indicates that substituents at position 3 of the pyridine ring would be ideal. Nicotine-mimicking compounds containing imidazole or methyl substituted imidazole side chains with varied sizes of 1–3 carbon alkyl spacers at position 3 of the pyridine ring were synthesized using Scheme 1 (compounds 1, 4 and 7). Based on our previous findings that acetylenic substituents can interact with the P450 active site amino acids and lead to mechanism-based inhibition, triple bond in the form of propargyl ether was incorporated in a second series of compounds at positions 2, 3 or 4 of the pyridine ring as an ether linkage (compounds 3, 6 and 2, respectively). Positions 2 and 4 were used to investigate whether substituted compounds at those positions would show a difference in their inhibition activities compared to the 3-position substituted compound. A longer spacer in between the propargyl ether and the 4-position of the pyridine ring was introduced to investigate whether a greater flexibility in the positioning of the key functional groups (pyridine ring and the alkyne moiety) in the binding site would be achieved. The Vivid CYP450 Screening Kit (Life Technologies, catalog #PV6140) was used for the P450 2A6 inhibition assays. An initial high-throughput screening was performed at a 10 μM concentration followed by a dose-response curve determination. Compounds 1, 4 and 6, all three containing a substituent on position 3 of the pyridine ring, were found to have single digit micromolar IC_50_ values [Figure 2, Table 1]. Compounds 5 and 7 were found to have moderate inhibition activity, with compound 7, a 3-position substituted compound, showing better inhibition than the 4-substituted compound 5. Compound 2, a 4-position propargyl ether substituted compound, exhibited much lower inhibition, while compound 3, a 2-postion propargyl ether substituted compound, did not inhibit P450 2A6 enzyme activity.

**Figure 3.**
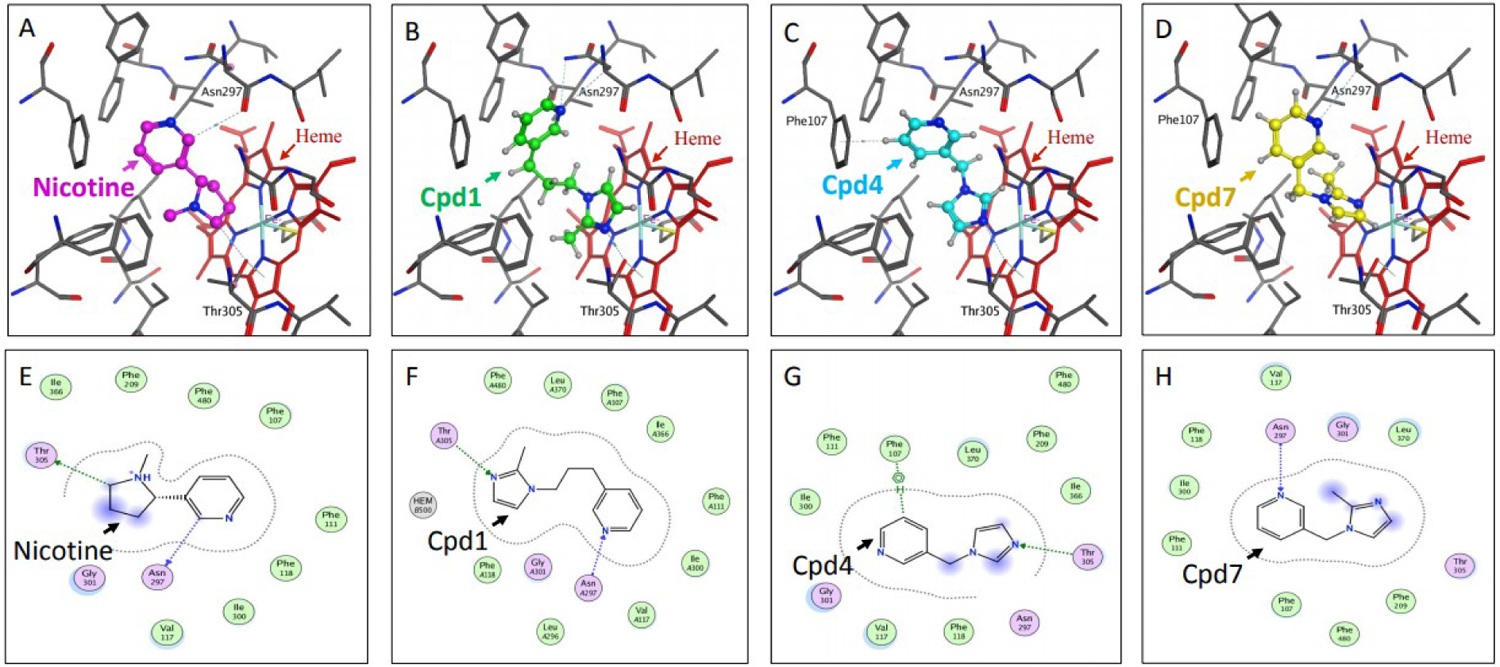
Docking studies on compounds (Cpd) that have pyridine with imidazole substituents (Cpd1, Cpd4 and Cpd7). Figures (A), (B), (C) and (D) show the binding modes and figures (E), (F), (G) and (H) show the ligand interactions with the active site residues for nicotine and compounds 1, 4 and 7, respectively in the active site of P450 2A6 enzyme.

**Figure 4.**
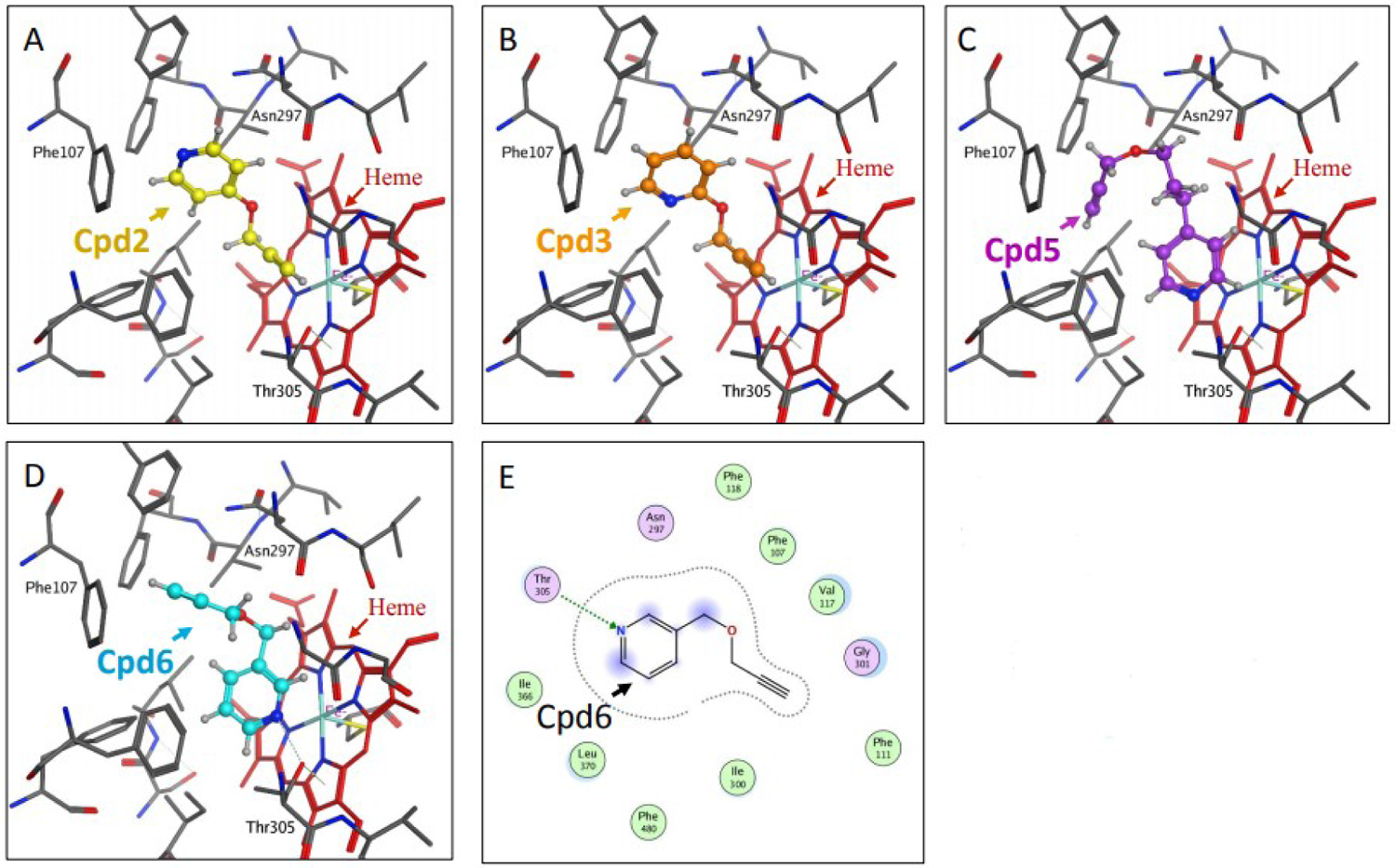
Docking studies on compounds (Cpd) that have pyridine with propargyl ether substituents (Cpd2, Cpd3, Cpd5 and Cpd6). Figures (A), (B), (C) and (D) show the binding modes of compounds 2, 3, 5 and 6, respectively with the active site of P450 2A6. Figure E depicts the ligand interactions of compound 6 with the active site residues.

Docking studies of compounds 1 through 7 with the reported X-ray crystal structure of P450 2A6 with nicotine in its active site (4EJJ.pdb) were conducted using the MOE software platform from the ChemComp group. The nicotine molecule makes two H-bonds with Thr305 and Asn297 [Figure 3A and E], and the −CH_2_ group of the pyrrolidine ring is in close proximity to the heme-Fe atom. The binding poses of the imidazole substituted compounds 1, 4 and 7 showed that all three molecules made H-bonds with the active site residues. Similar to the substitution pattern of nicotine, the imidazole substituents of these compounds are connected with varying alkyl chain lengths (1 or 3 carbons) at position 3 of the pyridine ring. Compound 1 made two H-bonds with Thr305 and Asn297, and had the −CH of the imidazole ring in close proximity to the heme-Fe atom [Figure 3B and F]. Compound 4 made one H-bond with Thr305, and aromatic π-H interaction with Phe107 [Figure 3C and G]. The −CH group at position 2 of the imidazole ring was facing the heme-Fe atom. Compound 7 made one H-bond with Asn297, and the sp2 nitrogen atom of the imidazole ring was in close proximity to the heme-Fe atom [Figure 3D and H]. The presence of the methyl substituent at the 2-position of the imidazole ring in this compound caused a change in the orientation of the methyl group, away from the heme-Fe atom, causing a flip in the positioning of this ring in the active site. The propargyl ether pyridine compounds 2, 3, 5 and 6 showed variations in binding poses, based on the position of the substituent [Figure 4]. Compounds 2 and 5 had substituents at position 4 of the pyridine ring. Compound 2 did not have a linker alkyl chain connecting the propargyl ether group to the pyridine ring [Figure 4A]. Compound 2 did not make any H-bond interactions with the active site residues, and the alkyne carbons were in close proximity to the heme-Fe atom. Compound 5 with a 3-carbon alkyl chain linker between the propargyl ether group and the pyridine ring depicted a flipped binding pose with the pyridine ring oriented towards the heme-Fe [Figure 4C]. Compound 3 with the propargyl ether group at position 2 of the pyridine ring depicted a binding pose identical to that of compound 2, with the alkyne carbons facing the heme-Fe atom [Figure 4B]. Compound 6 with the propargyl ether group connected by a methylene linker to position 2 of the pyridine ring had a binding pose similar to that of compound 5, with the pyridine ring facing the heme-Fe atom [Figure 4D and E]. Compound 6 was the only propargyl ether derivative to make a H-bond with the active site residue Thr305. The P450 2A6 inhibition studies clearly confirmed these observations and indicated that substitution at position 3 of the pyridine ring was the most ideal. The docking studies showed that the close proximity of the heterocyclic rings to the heme-Fe atom was most favored, and increases in H-bond interactions with the active site residues increased the inhibition potency.

The inhibition studies on our compounds were performed using a Vivid CYP2A6 Kit (Life Technologies, catalog #PV6140). This kit uses microsomes from insect cells stably expressing human CYP2A6 enzyme. As such all the enzyme from these microsomes is essentially CYP2A6, eliminating the possibility of the presence of other polymorphic variants of CYP2A6 that could be naturally found in liver microsomes. Therefore, the Vivid CYP2A6 Kit used in this study does not account for any polymorphism of CYP2A6. However, it is very important to understand the inhibitory effect of new compounds on CYP2A6 polymorphism as people with other allelic variants of CYP2A6 exhibit varied metabolic response. Our future studies would aim to study this phenomenon, either by developing the cell systems expressing various CYP2A6 allelic variants using baculovirus and insect cell lines by genetic engineering tools^[59–61]^, or by deploying the “molecular lego” approach developed by Castrignano *et al.*^[50]^, using a chimeric CYP2A6-flavodoxin/CYP2A6 and determining the kinetic parameters of coumarin electrocatalysis by electrochemical detection.

## DISCUSSION

Based on the pyridine scaffold of nicotine, two series of compounds were designed and synthesized for this study. The compounds containing an imidazole or propargyl ether substituents at position 3 of the pyridine ring were found to be promising lead compounds for further development. Our studies clearly illustrated that H-bonding interactions were very important for effective binding of these molecules within the P450 2A6 active site. Many of the pyridine derivatives developed by the Cashman Group have a primary or secondary amine functional group that interacts with the heme iron^[51–54]^. The compounds developed by our group do not contain any amine functional groups, resulting in the aromatic heterocyclic rings facing the heme-Fe and also showing low P450 2A6 inhibition IC_50_ values (micromolar). Using these lead compounds, additional potential inhibitors with increased number of interactions with the amino acid residues of the P450 2A6 active site will be designed and synthesized.

## Figures and Tables

**Table 1.** IC_50_ values of P450 2A6 inhibition by compounds 1 to 7

Compound	IC50 (μM)
1	9.034
2	193.8
3	0
4	5.024
5	66.91
6	1.003
7	42.51
